# Narrow band imaging complements eosinophilic esophagitis reference score in predicting inflammatory infiltration in patients with dysphagia

**DOI:** 10.1055/a-2685-7610

**Published:** 2025-09-09

**Authors:** Kotryna Truskaite, Laura Vossen Engblom, Greger Lindberg, Aldona Dlugosz

**Affiliations:** 1 27106 Department of Medicine at Huddinge, Karolinska Institute, Stockholm, Sweden; 2 59562 Division of Gastroenterology, Department of Gastroenterology, Dermatovenereology and Rheumatology, Karolinska University Hospital, Stockholm, Sweden; 3 27106 Center for Bioinformatics and Biostatistics, Karolinska Institute, Stockholm, Sweden

**Keywords:** Endoscopy Upper GI Tract, Eosinophilic esophagitis, Benign strictures, Diagnosis and imaging (inc chromoendoscopy, NBI, iSCAN, FICE, CLE)

## Abstract

**Background and study aims:**

Magnifying endoscopy with narrow-band imaging (ME-NBI) is regularly used in neoplasia diagnostics although its use in assessment of esophageal inflammatory changes is uncommon. The aim of this study was to evaluate the efficacy of eosinophilic esophagitis reference score and ME-NBI signs in predicting inflammation using gastroscopy with dual focus in patients with dysphagia.

**Patients and methods:**

We conducted a prospective cohort study in adults undergoing gastroscopy with esophageal biopsies because of dysphagia/food bolus impaction. Number of eosinophiles and lymphocytes were calculated per high-power field. We used logistic regression with forward stepwise selection to determine the most relevant predictors (endoscopic signs) of inflammation. To assess the predictive value of endoscopic signs for eosinophilic or lymphocytic infiltration, we calculated sensitivity, specificity, and predictive values.

**Results:**

In total 219 patients (71.2% male) were enrolled to the study. Most frequent endoscopic findings were furrows (121/219, 55%), positive NBI signs (106/219, 48%), and edema (102/219, 47%). Logistic regression analysis showed that furrows and NBI signs were the most significant predictors of eosinophilic infiltration. Edema was the only significant predictor of lymphocyte infiltration.

**Conclusions:**

Positive NBI signs and furrows were the best predictors of eosinophile infiltration, whereas lymphocytic infiltration was predicted by edema. Given that NBI is already widely available, we encourage use of both white light and NBI in patients with suspected esophageal inflammation.

## Introduction

Inflammatory disorders of the esophagus such as eosinophilic esophagitis (EoE) and lymphocytic esophagitis (LyE) 1 2 are common benign causes of dysphagia and/or food bolus impaction (FBI). Histologically EoE is defined as an eosinophil (eos) count ≥ 15 per high-power field (hpf) in the esophageal mucosa whereas intraepithelial lymphocytes (lympf) ≥ 40 per hpf is considered indicative of LyE. EoE is found in 12% to 22% of patients undergoing upper endoscopy for dysphagia 3 and accounts for over 50% of esophageal food impactions in young adults 3 4 . Our previous study showed that LyE was the cause of FBI in 9% of cases 4 .

Up to 96% of patients with EoE have at least one abnormal macroscopic finding upon upper endoscopy 5 6 7 . There is no single pathognomonic endoscopic sign for EoE that would indicate active inflammation histologically. Esophageal biopsy sampling is mandatory to confirm EoE diagnosis and/or response to treatment but because of the patchy nature of EoE, targeted biopsies based on visible inflammation are crucial. Endoscopic assessment of esophageal signs of EoE (edema, rings, furrows, exudates, stricture) known as Endoscopic Reference Score (EREFS) were introduced by Hirano et al. in 2013 8 and is now routinely used by endoscopists. LyE endoscopic features may present similarly to EoE but one-third of LyE patients have macroscopically normal mucosa 2 . To date, EREFS has not been validated for LyE and no studies have examined correlations between endoscopic and histologic findings in LyE.

Narrow-band imaging (NBI) and near-focus (NF) magnification enhances mucosal visualization and is widely used to detect subtle mucosal lesions. Our research group previously described esophageal signs revealed by magnification endoscopy with NBI (ME-NBI) that are associated with both EoE and LyE 9 . To our knowledge, no prospective studies have evaluated the diagnostic utility of ME-NBI signs using NBI instrument with NF mode.

The main aim of this study was to establish whether ME-NBI criteria using instrument with NF mode are informative for diagnosing esophageal inflammatory activity in patients with dysphagia symptoms.

Specific aims of this study were to test which endoscopic sign(s)—the EREFS signs and/or the ME-NBI signs itself—best predict(s) an elevated eosinophil and lymphocyte cell count revealed upon histological examination of biopsy specimens. Specifically, are previously developed ME-NBI criteria 10 informative and can they improve the diagnostic procedure for EoE and LyE in patients with dysphagia, compared with an evaluation consisting only of the standard EREFS signs?

## Patients and methods

The study was approved by the Regional Ethical Review Board of Stockholm, Sweden (Dnr: 2013/2269–31/4).

### Study population

This was a prospective cohort study of adult patients with dysphagia and/or FBI conducted at Karolinska University Hospital, Huddinge, Stockholm, Sweden and Stockholm Gastro Center, Stockholm, Sweden between January 2013, and December 2018. Two hundred nineteen patients referred for endoscopy because of dysphagia /FBI were consecutively recruited to our study. Patients with cancer or previous esophageal surgery were excluded.

### Endoscopy equipment and procedure

All patients underwent conventional white light endoscopy (WLE) followed by NBI and near-focus (NF-NBI). Endoscopic examinations were performed by single expert endoscopist (AD) using a GIF HQ190 Evis Exera III endoscope (Olympus, Tokyo, Japan) with dual focus (DF) capacity. Except for knowing the indication for gastroscopy, the endoscopist was blinded to patient information and previous endoscopy findings. Six biopsies, two from each level (distal, middle, and proximal) in the esophagus were obtained for histological assessment under NF-NBI guidance.

### Histopathologic analysis of biopsy specimens

Experienced pathologists analyzed all biopsies according to standard routines. Biopsies were assessed for the count of eosinophils using chromatic staining and the count of lymphocytes using immunohistochemistry for the pan-T-cell marker CD3 according to standard protocols. The hpf was defined as an area of 0.24 mm² with 400 times magnification. Eosinophils and lymphocytes were counted in the most densely infiltrated areas and the highest peak eosinophil and lymphocyte count number was recorded. Presence of ≥ 15 eosinophiles/hpf in the biopsy sample was considered elevated and use as the cut-off value for eosinophilic infiltration count whereas presence of ≥ 40 intraepithelial lymphocytes/hpf was considered elevated and was the cut-off value for lymphocytic infiltration, in line with current clinical standards 10 . Endoscopic findings were documented with still images and together with histopathological assessment stored in medical records.

### Study definitions

During gastroscopy we used the EREFS classification and grading system (EREFS) 8 . Edema and stenosis were recorded as present (1) or absent (0). White exudates (0–2), mucosal rings (0–3), and linear furrows (0–2) were subcategorized into grades according to Hirano et al. 8 but were not scored separately in the proximal, middle, or distal esophagus. From the above five EREFS signs three compound endoscopic scores were calculated: fibrotic sign score (sum of scores for rings and strictures), inflammatory sign score (sum of scores for exudates, furrows and edema), as well as total EREFS score (sum of scores for all five endoscopic signs, ranges from 0 to 9) 7 .

**Fig. 1:**
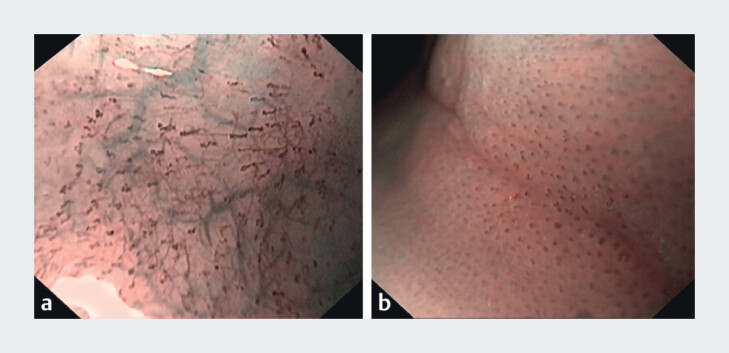
Endoscopic images of the esophagus using NF-NBI. **a** Normal esophagus. **b** Positive NBI criteria in patient with EoE: 1) beige mucosa (beige color of the mucosa, compared with normal mucosa, which has a light green colour); 2) dot IPCL (increased and dot-shaped congested IPCLs); and 3) absent cyan vessels (invisibility of cyan-coloured vessels that are normally found in the esophagus mucosa).

For NBI evaluation we used a combination of three criteria previously identified as indicative of EoE and LyE using magnifying endoscope. In our study we used a NF setting NBI to assess NBI signs ( Fig. 1 ) 9 . Each NBI criterion was described as absent or present. Cases with all three NBI signs were classified as NBI positive, whereas those lacking one or more criteria were deemed as NBI negative.

### Statistics

Statistical analyses were conducted in R statistical programming software version 4.2.1 11 . To assess validity of the endoscopic signs as diagnostic tests for eosinophilic or lymphocytic infiltration, we calculated values of sensitivity, specificity, positive predictive value (PPV), and negative predictive value (NPV) for all six endoscopic signs (recoded as binary variables) using the epiR package 12 . Eosinophil count (as a binary variable, < 15 or ≥ 15) was taken as the gold standard for eosinophilic infiltration and lymphocyte count (as a binary variable, < 40 or ≥ 40) was considered the gold standard for lymphocytic infiltration.

To assess how well each endoscopic sign by itself predicted eosinophilic/lymphocytic infiltration, we fitted univariate logistic regression models for each sign and outcome. To identify which of these signs best predicted each type of infiltration, we fitted two multivariable logistic regression models (one for eosinophilic and one for lymphocytic infiltration) containing all six endoscopic signs as predictor variables, which we termed the “full model”. In addition we used logistic regression with automatic forward stepwise variable selection based on Aikake Information Criterion (AIC; using the stats::step command 13 ) to create a model that only contained those endoscopic signs that best predicted eosinophilic/lymphocytic infiltration. For all logistic regression models, we ran residual diagnostics using the R package DHARMa 14 , which confirmed that all model assumptions were met.

## Results

Patient characteristics are summarized in Table 1 . In total, 219 patients were included in the study, of whom 71% were male and the median age was 40 years (range: 18 to 89 years). We identified 85 patients with eosinophilic infiltration (≥ 15 cells/hpf) and 76 patients with lymphocytic infiltration (≥ 40 cells/hpf). Forty-three patients had both eosinophilic and lymphocytic infiltration. Distribution of patient data is shown in **Supplementary Fig. 1** . All 219 patients were divided into separate groups depending on final diagnosis to compare described endoscopic signs with histologic outcome. Distribution of eosinophil and lymphocyte count depending on different final diagnosis groups is shown in **Supplementary Fig. 2** .

**Table:** **Table 1** Patient characteristics.

	**All patients**	**Eosinophil count**		**Lymphocyte count**	
**Characteristic**	**N = 219**	**Elevated N = 85**	**Normal N = 134**	**Elevated N = 76**	**Normal N = 143**
Age, median (IQR)	40 (30, 54)	31 (26, 42)	46 (36, 58)	37 (30, 49)	42 (30, 56)
Gender					
F	63	18	45	20	43
M	156	67	89	56	100
EOS, median (IQR)	6 (0–26)	35 (23–63)	0 (0–4)	18 (2–45)	0 (0–18)
LYMPH, median (IQR)	14 (0–50)	40 (0–68)	0 (0–36)	55 (50–79)	0 (0–13)
Eosinophil count (hpf)					
Elevated	85			43	42
Normal	134			33	101
Lymphocyte count (hpf)					
Elevated	76	43	33		
Normal	143	42	101		
Diagnosis*					
EoE	132	81	51	57	75
GERD	46	4	42	0	46
LyE	19	0	19	18	1
Others†	22	0	22	1	21
Patient characteristics in all patients, in patient groups with normal or high eosinophil count and in patient groups with normal or high lymphocyte count. Numbers indicate numbers of patients or numbers of cells, if not otherwise indicated. Continuous variables are presented as median and interquartile range (IQR). *Final diagnosis was extracted from patient medical records. Eosinophilic esophagitis (EoE) was defined by presence of ≥ 15 eosinophils/hpf. Lymphocytic esophagitis (LyE) was defined by presence of ≥ 40 intraepithelial lymphocytes/hpf. †Other diagnoses – achalasia 3/22, esophageal dysmotility unspecified 3/22, esophageal functional disorder 1/22, dysphagia of unknown origin 15/22. EOS, eosinophil; EoE, eosinophilic esophagitis; GERD, gastroesophageal reflux disease; IQR, interquartile range.					

The EoE group included 61% of cases (81/132) with active EoE (eos > 15/hpf) and 38% (51/132) with EoE in histologic remission (eos < 15/hpf). Among EoE patients 22% of cases (29/132) responded to proton pump inhibitor treatment. In the group with gastroesophageal reflux disease (GERD), four patients had significant eosinophilic infiltration (≥ 15 eos/hpf) but in all cases, infiltration was observed only in the distal part of the esophagus. Lymphocyte infiltration < 40 lymph/hpf was found in 14 patients with a GERD diagnosis. Significant infiltration of lymphocytes was found in one patient with achalasia with a lymphocyte count of 50 lymph/hpf. One patient with LyE at the time of gastroscopy had no lymphocyte infiltration because the gastroscopy was performed after treatment.

**Fig. 2:**
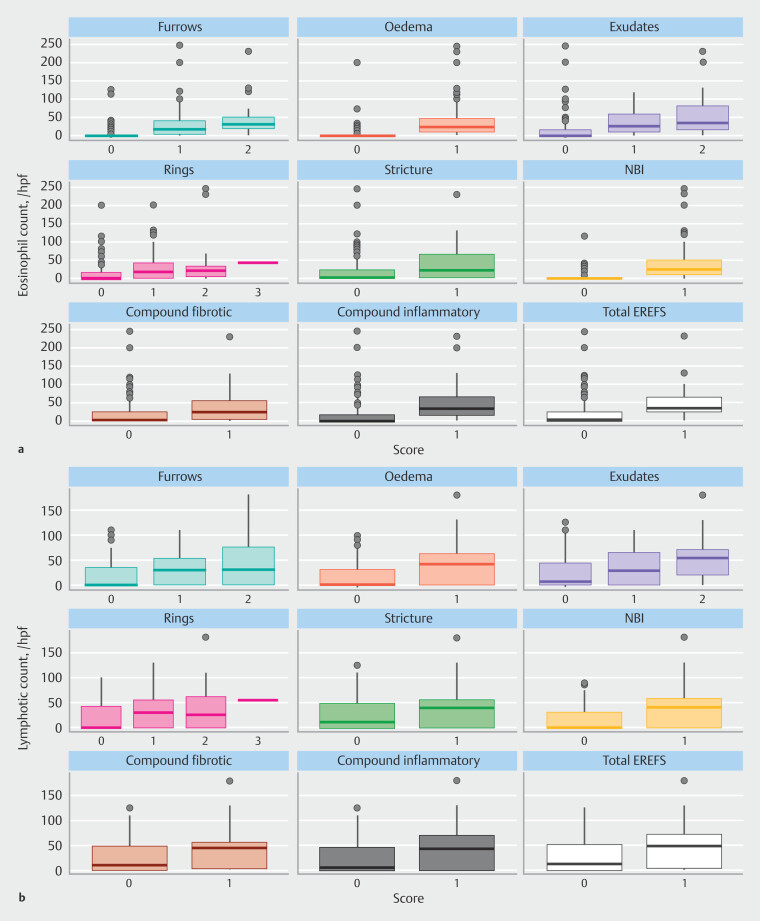
Eosinophil and lymphocyte count observed across the scores of the five EREFS signs and the single NBI sign. **a** Eosinophil cell count vs. EREFS/ NBI sign score. **b** Lymphocyte cell count vs. EREFS/ NBI sign score. Boxes display median and interquartile range (IQR). Whiskers extend to 1.5 times the IQR. Data beyond the whiskers are outliers.

Endoscopic signs and eosinophil/lymphocyte cell counts are summarized in Table 2 and Fig. 2 . Compound signs are summarized in **Supplementary Table 1** . The most frequent endoscopic signs observed in all patients were furrows (121/219, 55%), followed by NBI (106/219, 48%), edema (102/219, 47%), and rings (95/219, 43%). Exudates and strictures were present in 26% and 15% of patients, respectively. Compound fibrotic signs (rings and strictures) were present in 12% of patients (26/219). At least one positive inflammatory sign was found in 125 of 219 (57% of the study cases) and compound inflammatory signs were positive in 22% of patients (47/219). In 5% of patients (10/219) all EREFS endoscopic signs were positive whereas in 32% of patients (69/219), all EREFS endoscopic sign were absent.

**Table:** **Table 2** Endoscopic findings.

	**All patients**	**Eosinophil count**		**Lymphocyte count**	
**Characteristic**	**N = 219**	**Elevated N = 85**	**Normal N = 134**	**Elevated N = 76**	**Normal N = 143**
**Furrows**					
0	98	9	89	23	75
1	95	53	42	41	54
2	26	23	3	12	14
**Edema**					
0	117	15	102	22	95
1	102	70	32	56	48
**Exudates**					
0	160	41	119	46	114
1	42	29	13	18	24
2	17	15	2	12	5
**Rings**					
0	124	33	91	33	91
1	71	37	34	31	40
2	23	14	9	11	12
3	1	1	0	1	0
**Stricture**					
0	185	66	119	59	126
1	34	19	15	17	17
**NBI signs**					
0	113	12	101	21	92
1	106	73	33	55	51
Endoscopic findings in all patients, in patient groups with normal (< 15 cells/hpf) or elevated (≥ 15 cells/hpf) eosinophil count and in patient groups with normal (< 40 cells/hpf) or elevated (≥ 40 cells/hpf) lymphocyte count. Numbers indicate numbers of patients.					

NBI positivity was observed in 48% of patients (106/219); 52% (113/219) lacked characteristic signs. Among NBI-negative cases, three patients showed partial features without histological evidence of eosinophilic or lymphocytic infiltration: one with achalasia and one with GERD had only beige mucosa, whereas one PPI-treated EoE patient showed beige mucosa and absent cyan vessels.

### Diagnostic accuracy of EREFS and NBI signs

Table 3 and Table 4 display the values for sensitivity, specificity, PPV, and NPV for EREFS signs individually and compound signs (fibrotic, inflammatory and total), where eosinophil respectively lymphocyte infiltration were taken as the gold standard.

**Table:** **Table 3** Diagnostic accuracy of EREFS signs, compound signs (fibrotic, inflammatory, total EREFS) and the NBI sign as a marker for eosinophilic infiltration (defined as (≥ 15 cells/hpf)).

**Endoscopic sign**	**Sensitivity (95% CI)**	**Specificity (95% CI)**	**PPV (95% CI)**	**NPV (95% CI)**
Furrows	89 (81–95)	66 (58–74)	63 (54–71)	91 (83–96)
Exudates	52 (41–63)	89 (82–94)	75 (62–85)	74 (67–81)
Edema	82 (73–90)	76 (68–83)	69 (59–77)	87 (80–93)
Rings	61 (50–72)	68 (59–76)	55 (44–65)	73 (65–81)
Stricture	22 (14–33)	89 (82–94)	56 (38–73)	64 (57- 71)
Total EREFS (≥ 2)	93 (85–97)	67 (59–76)	65 (59–70)	94 (87–97)
Inflammatory (≥ 1)	98 (92–100)	76 (68–83)	68 (61–75)	98 (91–99)
Fibrotic (≥ 1)	79 (68–87)	48 (40–57)	45 (40–50)	81 (73–87)
NBI	86	(77–92)	75 (67–82)	69 (59–78)
CI, confidence interval; EREFS, Eosinophilic Esophagitis Reference Score; NBI, narrow band imaging; NPV negative predictive value; PPV, positive predictive value.				

**Table:** **Table 4** Diagnostic accuracy of EREFS signs, compound signs (fibrotic, inflammatory, total EREFS) and the NBI sign as a marker for lymphocytic infiltration (defined as (≥ 40 cells/hpf)).

**Endoscopic sign**	**Sensitivity (95% CI)**	**Specificity (95% CI)**	**PPV (95% CI)**	**NPV (95% CI)**
Furrows	70 (58–80)	52 (44–61)	44 (38–49)	77 (69–83)
Exudates	39 (28–51)	80 (72–86)	51 (40–61)	71 (67–75)
Edema	71 (60–81)	66 (58–74)	53 (46–59)	81 (75–86)
Rings	57 (45–68)	64 (55–72)	45 (38–53)	73 (67- 79)
Stricture	22 (14–33)	88 (82–93)	50 (35–64)	68 (65–71)
Total EREFS (≥ 2)	78 (67–86)	56 (47–64)	48 (43–54)	82 (75–88)
Inflammatory (≥ 1)	79 (68–87)	48 (40–57)	45 (40–50)	81 (73–87)
Fibrotic (≥ 1)	45 (35–55)	68 (61–75)	45 (37–52)	69 (65–73)
NBI	71 (59–81)	63 (54–70)	48 (42–54)	81 (75–87)
CI, confidence interval; EREFS, Eosinophilic Esophagitis Reference Score; NBI, narrow band imaging; NPV negative predictive value; PPV, positive predictive value.				

Of the individual endoscopic signs, furrows, NBI, and edema had the highest sensitivity (89%, 86%, and 82%, respecitvely) for correctly identifying patients with an elevated eosinophil count. Specificity of these signs was lower (66%, 75%, and 76%, respectively), indicating that even patients without eosinophilic infiltration sometimes presented with these signs. The same trend was observed for identifying patients with elevated lymphocyte count, where NBI and edema had a sensitivity of 71% and NPV of 81% but lower specificity: 63% and 66%, respectively.

Different cut-off values for compound EREFS sign scores were calculated ( **Supplementary Table 2** and **Supplementary Table 3** ). Total EREFS score ≥ 2 and inflammatory score ≥ 1 had a high sensitivity of 93% and 98% and lower specificity of 67% and 76%, respectively, predicting eosinophilic infiltration. None of the compound EREFS scores showed statistically significant predictive values for lymphocyte count. Fibrotic signs—rings and strictures separately or as a compound fibrotic sign—had low sensitivity, specificity, and predictive values for both eosinophil and lymphocyte count.

### Which endoscopic signs(s) predict(s) eosinophil infiltration?

Each endoscopic sign by itself significantly predicted eosinophilic infiltration. The full model and forward selected logistic regression models revealed that only two endoscopic signs best predicted eosinophilic infiltration: furrows ( *P* = 0.004) and NBI ( *P* < 0.001; Table 5 ). In addition, adding exudates to the model with furrows and NBI also improved model fit, but this sign was not statistically significant in the final forward selected model ( *P* = 0.076). Strictures ( *P* > 0.9), rings ( *P* = 0.3), and edema ( *P* = 0.14) did not significantly eosinophilic infiltration in the full model.

**Table:** **Table 5** Results of logistic regression with eosinophil count (as a binary variable, < 15 or ≥ 15) as outcome.

	**Univariable**				**Full model**			**Forward selected**		
**Endoscopic sign**	**N**	**OR**	**95% CI**	***P* value **	**OR**	**95% CI**	**P value**	**OR**	**95% CI**	***P* value **
Furrows	219			**< 0.001**			**0.003**			**0.004**
0		—	—		—	—		—	—	
1		12.5	5.87–29.3		3.68	1.27–11.0		3.32	1.19–9.40	
2		75.8	21.6–369		13.8	2.84–83.8		11.9	2.67–67.1	
Edema	219			< 0.001			0.14			
0		—	—		—	—				
1		14.9	7.69–30.4		2.20	0.76–6.25				
Exudates	219			**< 0.001**			0.12			0.076
0		—	—		—	—		—	—	
1		6.47	3.14–14.0		2.08	0.85–5.21		2.22	0.92–5.52	
2		21.8	5.82–142		3.70	0.78–27.0		4.01	0.88–28.7	
Rings	219			**< 0.001**			0.3			
0		—	—		—	—				
1		3.00	1.63–5.58		0.93	0.37–2.23				
2		4.29	1.72–11.2		0.48	0.13–1.77				
3		5,841,042	0.00-NA		4,179,207	0.00-NA				
Stricture	219			**0.028**			> 0.9			
0		—	—		—	—				
1		2.28	1.09–4.86		1.06	0.39–2.92				
NBI	219			**< 0.001**			**0.041**			**< 0.001**
0		—	—		—	—		—	—	
1		18.6	9.30–40.0		3.47	1.05–12.3		5.59	2.20–14.8	
AIC					201			197		
Predictor variables are the six endoscopic signs: furrows, oedema, exudates, rings, strictures (scored from 0 to 3 or 0 to 2) and NBI. In the univariable model, each sign is added as a single predictor in each model. The full model contains all six signs as predictors. The forward selected model only contains those endoscopic signs that increased the fit of the model significantly. AIC, Akaike Information Criterion – measures model fit, with a lower value indicating a better fit; CI, confidence interval; NBI, narrow band imaging; OR, odds ratio.										

We performed equivalent analyses using dichotomized endoscopic signs (coding each as present or absent instead of scores from 0 to 3 or 0 to 2). These models also identified NBI ( *P* <0.001) and furrows ( *P* = 0.008) as the most important predictors of eosinophil infiltration, and here exudates had a significant effect on eosinophilic infiltration ( *P* = 0.006; **Supplementary Table 4** ).

### Which endoscopic signs(s) predict(s) lymphocyte count?

The results using the full model and the forward stepwise selection model showed that edema ( *P* < 0.0001) was the only endoscopic sign that significantly predicted lymphocytic infiltration ( Table 6 ). Adding additional endoscopic signs did not improve the fit of this model. The same result was achieved within equivalent analyses using dichotomized variables rather than scores ( **Supplementary Table 5** ). Because there is no international consensus regarding significant lymphocyte infiltration threshold value, we performed logistic regression calculations using different lymphocyte cut-off values (30 cells/hpf or 20 cells/hpf; **Supplementary Table 6** and **Supplementary Table 7** ) and found no significant differences in results.

**Table:** **Table 6** Results of logistic regression with lymphocyte count (as a binary variable, < 40 or ≥ 40) as outcome.

	**Univariable**				**Full model**			**Forward selected**		
**Endoscopic sign**	**N**	**OR**	**95% CI**	***P* value **	**OR**	**95% CI**	***P* value **	**OR**	**95% CI**	***P* value **
Furrows	219			**0.006**			0.2			
0		—	—		—	—				
1		2.48	1.34–4.65		0.58	0.19–1.55				
2		2.80	1.13–6.93		0.29	0.07–1.16				
Edema	219			**< 0.001**			**0.045**			**< 0.001**
0		—	—		—	—		—	—	
1		4.86	2.68–9.04		2.95	1.02–8.94		4.86	2.68–9.04	
Exudates	219			**0.002**			0.084			
0		—	—		—	—				
1		1.86	0.91–3.74		1.01	0.45–2.26				
2		5.95	2.08–19.6		4.07	1.12–17.5				
Rings	219			**0.019**			0.6			
0		—	—		—	—				
1		2.14	1.16–3.97		1.42	0.66–3.04				
2		2.53	1.01–6.32		0.98	0.31–3.08				
3		5,841,042	0.00-NA		1,202,370	0.00-NA				
Stricture	219			**0.046**			0.8			
0		—	—		—	—				
1		2.14	1.02–4.50		1.13	0.47–2.67				
NBI	219			**< 0.001**			0.15			
0		—	—		—	—				
1		4.72	2.61–8.83		2.46	0.73–9.07				
AIC					265			258		
Predictor variables are the six endoscopic signs: furrows, oedema, exudates, rings, strictures (scored from 0 to 3 or 0 to 2) and NBI. In the nivariable model, each sign is added as a single predictor in each model. The full model contains all six signs as predictors. The forward selected model only contains those endoscopic signs that increased the fit of the model significantly. AIC, Akaike Information Criterion – measures model fit, with a lower value indicating a better fit; CI, confidence interval; NBI, narrow band imaging; OR, odds ratio.										

## Discussion

Our data show that eosinophilic infiltration is best predicted by two signs: NBI and furrows. Adding more EREFS signs (exudates, edema, rings and strictures) did not improve the predictive models. Although each sign by itself significantly predicted eosinophilic infiltration in univariate models, only one EREFS sign remained significant when all signs were added to the model. This suggests that the information provided by the five EREFS signs overlaps whereas the NBI sign seems to capture other aspects of eosinophilic infiltration that are not captured by the EREFS signs. With regard to lymphocytic infiltration, we found that it was predicted best by only one EREFS sign: edema.

NBI with or without magnification has been studied before in the context of EoE and LyE. Peery et al used conventional endoscopy and NBI without magnification or definition of specific NBI signs to detect furrows, exudates, and rings and found that diagnostic rate in EoE patients did not improve using NBI 15 . Retrospective analysis published by Ichiya et al suggested that evaluation of NBI signs with magnification (magnifying endoscopes) together with conventional endoscopy improved detection of both EoE and LyE 16 . A recently published study by Ayaki M. et al. 17 evaluated beige color of mucosa (one of the NBI signs) and found that presence of this special NBI sign can indeed accurately identify histologically active sites of inflammation in patients with EoE. The authors concluded that NBI is useful for recognizing inflammation in areas without morphological changes 17 . Their data showed 97.7% sensitivity, 96.9% specificity, and 97.8% overall accuracy for beige mucosa in predicting eosinophilic esophagitis activity (eos > 15 eos/hpf). We combined conventional endoscopy with NF NBI and used specific NBI signs for evaluation of endoscopic changes in esophagus. In our study NBI was associated with eosinophilic infiltration with high sensitivity (86%) and high NPVs (89%). NBI regarding high lymphocyte count had high NPV (81%) and lower sensitivity (71%) and specificity (63%). Our results show that NBI signs are good indicators of eosinophilic and lymphocytic inflammation but are more specific and accurate for evaluating histologic active inflammation where eosinophilic infiltration dominates. Overall, we believe that use of magnification and identification of specific NBI signs is of significant importance in endoscopic evaluation of patients with dysphagia.

We found that furrows, edema, and rings were the most frequently detected endoscopy signs and they were more specific for eosinophilic esophagitis compared with lymphocytic esophagitis. Exudates were the only endoscopic sign with PPVs and NPVs (75% and 74%, respectively) regarding eosinophilic infiltration, but not lymphocytic infiltration. We concluded that absence of furrows and edema would indicate low count of both eosinophils and lymphocytes. Strictures were found to be the least significant endoscopic sign for predicting inflammatory changes. We found low predictive values for both individual and compound fibrotic endoscopic signs, a result that corresponds with other publications. Absence of an association between fibrotic signs and histologically active disease was described by several authors 7 18 , who found that rings and strictures were not consistent with eosinophilic infiltration. Moreover, these signs were often observed even after remission of active EoE had been achieved.

Dellon et al showed a significant utility of composite endoscopic scoring for prediction of EoE status (active vs remission of EoE) and found that total EREFS score ≥ 2 had the best correlation with mucosal eosinophilia. These results correspond with our findings where the optimal cut point was ≥ 2 for total EREFS score to predict eosinophilic infiltration. Moreover, according to Dellon et al. and Van Rhijn et al. 19 EREFS inflammatory score (edema, furrows, and exudates) was even better than total EREFS or predicting active EoE status. However, it should be noted that the authors did not report the exact cut-off values used, which can influence values obtained for sensitivity, specificity, PPV, and NPV, thereby making exact comparisons between studies difficult. We found that the optimal cut point for inflammatory EREFS score was ≥ 1 with sensitivity of 98%, specificity of 76%, and NPV 98%.

In the meta-analysis by Kim et al. 6 , overall sensitivity, specificity, pooled PPV, and NPV was assessed for each separate endoscopic finding. Their results showed that 83% of EoE cases had at least one endoscopic abnormality; however, the predictive value of individual endoscopic signs was too low to indicate EoE. In our results, separate WLE signs did not exhibit high sensitivity on their own but diagnostic power became higher if they were classified into inflammatory and total EREFS. All signs should be evaluated concurrently to predict the histologic outcome.

Lymphocyte infiltration in the esophagus and its correlation with endoscopic signs has not been well studied. We found that 47% of cases (9/19) of LyE had no endoscopic signs of disease. This finding is consistent with published literature about endoscopic appearance of LyE patients. Endoscopic features in LyE patients are very heterogeneous and one-third to one-half of LyE patients have no endoscopic abnormalities 20 21 . It is widely reported that endoscopic appearance of LyE patients is analogous and often macroscopically indistinguishable from EoE 22 23 . Tanaka et al found that 72.7% of LyE patients in a studied cohort had at least one of the WLE signs that are commonly found in EoE patients 9 . The most common endoscopic features in LyE according to a systematic review published by Habbal et al *.* were erosive esophagitis, rings, strictures, and furrows 20 . We found that 53%of LyE patients (10/19) had at least one positive endoscopic sign (most commonly rings, furrows, edema), 37% (7/19) were NBI positive and that edema was the only endoscopic sign with the strongest predictive value on lymphocyte infiltration. Our data regarding lymphocytic infiltration showed that combined EREFS signs were not sufficient to predict lymphocytic inflammation because total, inflammatory, and fibrotic EREFS signs scores had high NPVs (82%, 78%, and 69%, respectively) and low PPVs (48%, 51%, and 45%, respectively).

The finding that EoE and LyE shared similar endoscopic appearance and clinical symptoms of dysphagia raises the question whether LyE is a separate disease entity or a phenotype variation of EoE. Recently a multicenter study characterized LyE as a variant of EoE on the basis of mRNR sequencing, which showed the same genes were upregulated in LyE as in EoE 24 . Further studies and preferably an international consensus is needed to determine the relationship between EoE and LyE.

In our cohort, significant lymphocytic infiltration (> 40/hpf) was found in 76 of 219 cases (34%), including 56 cases with EoE, 18 with LyE, and one with achalasia. Dysmotility disorders can exhibit both eosinophilic and lymphocytic infiltration 25 26 . Although eosinophils are the primary histological marker of EoE, other cell types such as mast cells and lymphocytes have been also identified, but their exact role and significance in EoE pathogenesis remains unclear 27 . Our cohort included 43 of 219 cases with both significant eosinophilic and lymphocytic infiltration. As our study results show, both inflammatory cell types (lymphocytes and eosinophils) can be found in cases with positive EREFS and NBI endoscopic signs; however, it remains uncertain whether the observed endoscopic signs are attributable to eosinophils, lymphocytes, or both. Further studies are needed to clarify the pathogenetic role of lymphocytes in EoE.

As far as we know, our prospective study is the largest that has assessed associations between endoscopic signs and histologic findings (both eosinophil and lymphocyte counts) in patients with dysphagia. Moreover, we included cases of eosinophilic esophagitis responsive to PPI therapy (previously termed PPI-REE) a population often excluded in earlier research. The prospective design and inclusion of a broad patient spectrum are the key strengths of this study, enhancing generalizability of our findings. However, absence of internationally accepted diagnostic criteria for lymphocytic esophagitis and thresholds for significant lymphocytic infiltration remains a major limitation because it restricts comparability of results across studies.

## Conclusions

In conclusion, our study provides new evidence that NBI assessment with NF mode—an endoscopy tool that is now accessible in many endoscopy centers—should be used to evaluate dysphagia patients because NBI criteria used together with WLE add more evidence predicting presence of inflammation and make the diagnostic procedure easier. Endoscopic signs such as furrows, edema, and positive NBI signs are good indicators of the inflammatory process in esophageal mucosa and their presence should be enough to facilitate decision-making about use of anti-inflammatory treatment immediately after diagnostic endoscopy. This direct treatment strategy should be studied more closely in the future.

## Supplementary Material

Supplementary Material Zusatzmaterial
