# Supplementary material for: Narrow band imaging complements eosinophilic esophagitis reference score in predicting inflammatory infiltration in patients with dysphagia
Source: Endosc Int Open. 2025 Sep 9;13:a26857610. doi: 10.1055/a-2685-7610 (PMC12445250; doi:10.1055/a-2685-7610)
Supplement: Supplementary file 1 — Supplementary Material [file 10-1055-a-2685-7610_26875024.pdf]

Supplementary Table 1 Fibrotic-inflammatory and total EREFS scores in all patients (column 2) in patients with normal or elevated eosinophil count (columns 3 and 4) and in patients with normal or elevated lymphocyte count (columns 5 and 6).

	**All patients**	**Eosinophil count**		**Lymphocyte count**	
**Characteristic**	**N = 219**	**Elevated- N = 85**	**Normal- N = 134**	**Elevated- N = 76**	**Normal- N = 143**
**Compound fibrotic signs**					
0	116	29	87	30	86
1	65	33	32	26	39
2	26	16	10	14	12
3	11	6	5	5	6
4	1	1	0	1	0
**Compound inflammatory signs**					
0	85	2	83	16	69
1	33	14	19	8	25
2	44	24	20	22	22
3	33	22	11	17	16
4	15	15	0	7	8
5	9	8	1	6	3
**Total EREFS**					
0	69	2	67	15	54
1	28	4	24	2	26
2	28	14	14	7	21
3	32	18	14	18	14
4	33	26	7	19	14
5	15	9	6	7	8
6	9	7	2	5	4
7	2	2	0	1	1
8	3	3	0	2	0

EREFS-Eosinophilic Esophagitis Reference Score.

Endosc Int Open | DOI 10.1055/a-2685-7610 | © The Author(s).

Supplementary Table 2 Diagnostic accuracy of different cut-off values for EREFS compound signs (total EREFS-inflammatory and fibrotic) as a marker for eosinophilic infiltration (defined as (≥ 15 cells/hpf)).

**Total EREFS threshold**	**Sensitivity (95% CI)**	**Specificity (95% CI)**	**PPV (95% CI)**	**NPV (95% CI)**
≥ 1	98 (92-100)	50 (41-59)	55 (51-60)	97 (89-99)
≥ 2	93 (85-97)	67 (59-76)	65 (59-70)	94 (87-97)
≥ 3	76 (66-85)	78 (70-85)	69 (61-76)	84 (78-89)
≥ 4	55 (44-66)	89 (82-94)	76 (65-84)	76 (71-80)
≥ 5	23 (15-35)	94 (89-97)	72 (55-85)	66 (63-69)
≥ 6	14 (7-23)	99 (95-100)	86 (58-96)	64 (62-66)
≥ 7	6 (2-13)	100 (97-100)	100 (48-100)	63 (61-64)
≥ 8	4 (1-10)	100 (97-100)	100 (29-100)	62 (61-63)
**Inflammatory EREFS threshold**				
≥ 1	98 (92-100)	76 (68-83)	68 (61-75)	98 (91-99)
≥ 2	81 (71-89)	62 (53-70)	62 (57-67)	86 (80-91)
≥ 3	53 (42-64)	91 (85-95)	79 (68-87)	75 (71-79)
≥ 4	27 (18-38)	92 (87-96)	68 (52-80)	68 (65-71)
≥ 5	9 (4-18)	99 (96-100)	89 (50-98)	63 (62-65)
**Fibrotic EREFS threshold**				
≥ 1	66 (55-76)	65 (56-73)	54 (47-61)	75 (68-81)
≥ 2	27 (18-38)	89 (82-94)	61 (46-73)	66 (62-69)
≥ 3	8 (3-16)	96 (92-99)	58 (31-81)	62 (61-64)
≥ 4	1 (0-6)	100 (97-100)	100 (2-100)	61 (61-62)

EREFS, Eosinophilic Esophagitis Reference Score.

Endosc Int Open | DOI 10.1055/a-2685-7610 | © The Author(s).

Supplementary Table 3 Diagnostic accuracy of different cut-off values for EREFS compound signs (total EREFS-inflammatory and fibrotic) as a marker for lymphocytic infiltration (defined as (≥ 40 cells/hpf))

**Total EREFS threshold**	**Sensitivity (95% CI)**	**Specificity (95% CI)**	**PPV (95% CI)**	**NPV (95% CI)**
≥ 1	80 (70-89)	38 (30-46)	41 (37-45)	78 (69-86)
≥ 2	78 (67-86)	56 (47-64)	48 (43-54)	82 (75-88)
≥ 3	68 (56-79)	71 (62-78)	55 (48-62)	74 (83-86)
≥ 4	45 (33-57)	80 (73-87)	55 (44-65)	73 (69-77)
≥ 5	20 (11-30)	90 (84-95)	52 (35-68)	68 (65-71)
≥ 6	11 (6-20)	96 (91-98)	57 (32-79)	67 (65-69)
≥ 7	4 (1-11)	99 (95-100)	60 (20-90)	66 (65-67)
≥ 8	3 (0-9)	99 (96-100)	67 (16-96)	66 (65-67)
**Inflammatory EREFS threshold**				
≥ 1	79 (68-87)	48 (40-57)	45 (40-50)	81 (73-87)
≥ 2	68 (57-79)	66 (57-73)	51 (45-58)	78 (73-85)
≥ 3	39 (28-51)	81 (74-87)	53 (42-63)	72 (67-75)
≥ 4	17 (9-27)	92 (87-96)	54 (36-71)	68 (65-70)
**Fibrotic EREFS threshold**				
≥1	45 (35-55)	68 (61-75)	45 (37-52)	69 (65-73)
≥2	26 (16-38)	87 (81-92)	53 (38-66)	69 (66-72)
≥3	8 (3-16)	96 (91-98)	50 (25-75)	66 (65-68)
≥4	1 (0-7)	100 (97-100)	100 (2-100)	66 (65-66)

CI, confidence interval; EREFS, Eosinophilic Esophagitis Reference Score; NPV, negative predictive value; PPV, positive predictive value.

Endosc Int Open | DOI 10.1055/a-2685-7610 | © The Author(s).

Supplementary Table 4 Results of logistic regression models with eosinophil count (as a binary variable-< 15 or ≥ 15) as outcome.

		Univariable			Full model			Forward selection		
**Endoscopic sign**	**N**	**OR**	**95% CI**	***P* value**	**OR**	**95% CI**	***P* value**	**OR**	**95% CI**	***P* value**
Furrows	219			**< 0.001**			**0.012**			**0.008**
0		—	—		—	—		—	—	
1		16.7	8.02-38.6		3.61	1.33-10.1		3.83	1.42-10.6	
Edema	219			**< 0.001**			0.082			
0		—	—		—	—				
1		14.9	7.69-30.4		2.49	0.89-6.88				
Exudates	219			**< 0.001**			**0.014**			**0.006**
0		—	—		—	—		—	—	
1		8.51	4.38-17.3		2.74	1.22-6.28		3.03	1.36-6.89	
Rings	219			**< 0.001**			0.8			
0		—	—		—	—				
1		3.33	1.90-5.93		0.91	0.38-2.08				
Stricture	219			**0.028**			> 0.9			
0		—	—		—	—				
1		2.28	1.09-4.86		1.02	0.40-2.70				
NBI	219			**< 0.001**			**0.042**			**< 0.001**
0		—	—		—	—		—	—	
1		18.6	9.30-40.0		3.35	1.05-11.3		5.80	2.36-14.9	
AIC					202			199		

Predictor variables are the six endoscopic signs: furrows-edema-exudates-rings-strictures (scored from 0 to 3 or 0 to 2)-and NBI. In the univariable model-each sign is added as a single predictor in each model. The full model contains all six signs as predictors. The forward selected model only contains those endoscopic signs that increased the fit of the model significantly.

Endosc Int Open | DOI 10.1055/a-2685-7610 | © The Author(s).

AIC, Akaike Information Criterion – measures model fit-with a lower value indicating a better fit; CI, confidence interval; NBI, narrow band imaging; OR, odds ratio.

Endosc Int Open | DOI 10.1055/a-2685-7610 | © The Author(s).

Supplementary Table 5 Results of logistic regression with lymphocyte count (as a binary variable-< 40 or ≥ 40) as outcome.

		**Univariable**			**Full model**			**Forward selection**		
**Endoscopic sign**	**N**	**OR**	**95% CI**	***P* value**	**OR**	**95% CI**	***P* value**	**OR**	**95% CI**	***P* value**
Furrows	219			**0.001**			0.2			
0		—	—		—	—				
1		2.54	1.42-4.64		0.52	0.17-1.36				
Edema	219			**< 0.001**			0.059			**< 0.001**
0		—	—		—	—		—	—	
1		4.86	2.68-9.04		2.72	0.96-8.15		4.86	2.68-9.04	
Exudates	219			**0.003**			0.5			
0		—	—		—	—				
1		2.56	1.39-4.76		1.27	0.61-2.63				
Rings	219			**0.004**			0.7			
0		—	—		—	—				
1		2.28	1.30-4.05		1.13	0.54-2.29				
Stricture	219			**0.046**			0.8			
0		—	—		—	—				
1		2.14	1.02-4.50		1.12	0.48-2.57				
NBI	219			**< 0.001**			0.090			
0		—	—		—	—				
1		4.72	2.61-8.83		2.87	0.85-10.7				
AIC					263			258		

Predictor variables are the six endoscopic signs: furrows-edema-exudates-rings-strictures (scored from 0 to 3 or 0 to 2) and NBI. In the univariable model-each sign is added as a single predictor in each model. The full model contains all six signs as predictors. The forward selected model only contains those endoscopic signs that increased the fit of the model significantly.

AIC, Akaike Information Criterion – measures model fit-with a lower value indicating a better fit; CI, confidence interval; NBI, narrow band imaging; OR, odds ratio.

Endosc Int Open | DOI 10.1055/a-2685-7610 | © The Author(s).

Supplementary Table 6 Results of logistic regression with lymphocyte count (as a binary variable < 30 or ≥ 30) as outcome.

		Univariate			Full model			Forward selected		
**Endoscopic sign**	**N**	**OR**	**95% CI**	***P* value**	**OR**	**95% CI**	***P* value**	**OR**	**95% CI**	***P* value**
Furrows	219			**0.012**			0.4			
0		—	—		—	—				
1		2.31	1.29-4.20		0.79	0.31-1.93				
2		2.27	0.94-5.52		0.42	0.11-1.52				
Edema	219			**< 0.001**			0.15			**< 0.001**
0		—	—		—	—		—	—	
1		3.36	1.93-5.94		2.09	0.77-5.79		3.36	1.93-5.94	
Exudates	219			**0.011**			0.2			
0		—	—		—	—				
1		1.76	0.88-3.51		1.07	0.48-2.36				
2		4.22	1.49-13.8		3.44	0.96-14.2				
Rings	219			**0.018**			0.3			
0		—	—		—	—				
1		2.33	1.29-4.26		1.72	0.84-3.52				
2		1.86	0.75-4.59		0.93	0.30-2.83				
3		4-288-023	0.00-NA		1-607-249	0.00-NA				
Stricture	219			0.15			> 0.9			
0		—	—		—	—				
1		1.73	0.83-3.63		1.03	0.44-2.40				
NBI	219			**<0.001**			0.3			
0		—	—		—	—				
1		3.45	1.98-6.12		1.83	0.59-5.83				
AIC					291			283		

Predictor variables are the six endoscopic signs: furrows-edema-exudates-rings-strictures (scored from 0 to 3 or 0 to 2) and NBI. In the univariable model-each sign is added as a single predictor in each model. The full model contains all six signs as predictors. The forward selected model only contains those endoscopic signs that increased the fit of the model significantly.

AIC, Akaike Information Criterion – measures model fit-with a lower value indicating a better fit; CI, confidence interval; NBI, narrow band imaging; OR, odds ratio.

Endosc Int Open | DOI 10.1055/a-2685-7610 | © The Author(s).

Supplementary Table 7 Results of logistic regression with lymphocyte count (as a binary variable < 20 or ≥ 20) as outcome.

		**Univariate**			**Full model**			**Forward selected**		
**Endoscopic sign**	**N**	**OR[1]**	**95% CI**	***P* value**	**OR**	**95% CI**	***P* value**	**OR**	**95% CI**	***P* value**
Furrows	219			**0.003**			0.8			
0		—	—		—	—				
1		2.48	1.39-4.47		1.13	0.47-2.64				
2		3.01	1.25-7.57		0.81	0.22-2.93				
Edema	219			**< 0.001**			0.2			**< 0.001**
0		—	—		—	—		—	—	
1		3.24	1.87-5.69		2.00	0.74-5.50		3.24	1.87-5.69	
Exudates	219			**0.022**			0.2			
0		—	—		—	—				
1		1.45	0.73-2.89		0.82	0.37-1.80				
2		4.29	1.45-15.7		2.83	0.77-12.4				
Rings	219			**0.008**			0.2			
0		—	—		—	—				
1		2.52	1.39-4.62		1.84	0.91-3.74				
2		2.13	0.87-5.36		1.06	0.35-3.25				
3		3,470,210	0.00- NA		1,775,877	0.00-NA				

Endosc Int Open | DOI 10.1055/a-2685-7610 | © The Author(s).

		Univariate			Full model			Forward selected		
**Endoscopic sign**	**N**	**OR[1]**	**95% CI**	***P* value**	**OR**	**95% CI**	***P* value**	**OR**	**95% CI**	***P* value**
Stricture	219			0.15			➢ 0.9			
0		—	—		—	—				
1		1.72	0.82-3.67		1.03	0.44-2.42				
NBI	219			**< 0.001**			0.6			
0		—	—		—	—				
1		3.26	1.88-5.71		1.38	0.45-4.20				
AIC					298			289		

Predictor variables are the endoscopic signs: furrows, oedema, exudates, rings, strictures and NBI. Predictor variables are the six endoscopic signs: furrows, edema, exudates, rings, strictures (scored from 0 to 3 or 0 to 2) and NBI. In the univariable model, each sign is added as a single predictor in each model. The full model contains all six signs as predictors. The forward selected model only contains those endoscopic signs that increased the fit of the model significantly.
AIC, Akaike Information Criterion – measures model fit-with a lower value indicating a better fit; CI, confidence interval; NA, not available; NBI, narrow band imaging; OR, odds ratio.

Endosc Int Open | DOI 10.1055/a-2685-7610 | © The Author(s).

Figure S1. Histogram showing distribution of patient data: eosinophils and lymphocytes per hpf.

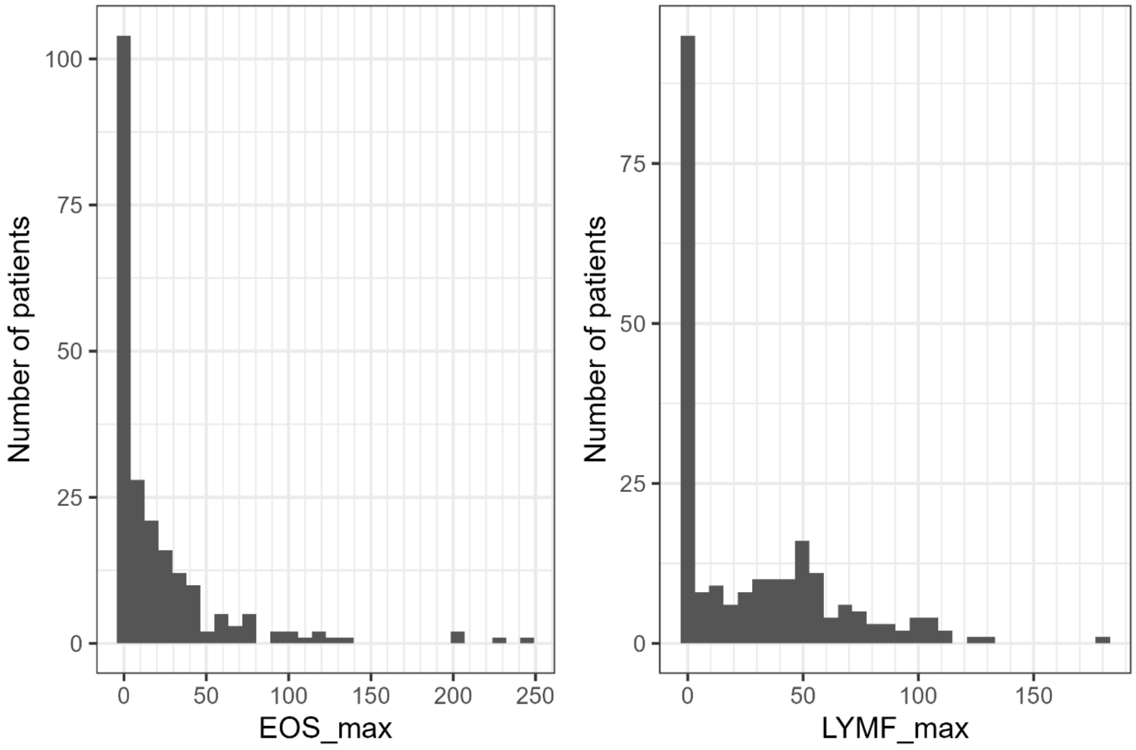

Endosc Int Open | DOI 10.1055/a-2685-7610 | © The Author(s).

Figure S2. Eosinophil and lymphocyte infiltration in different final diagnosis groups. Boxes display median and interquartile range (IQR). Whiskers extend to 1,5 times the IQR. Data beyond the whiskers are outliers.

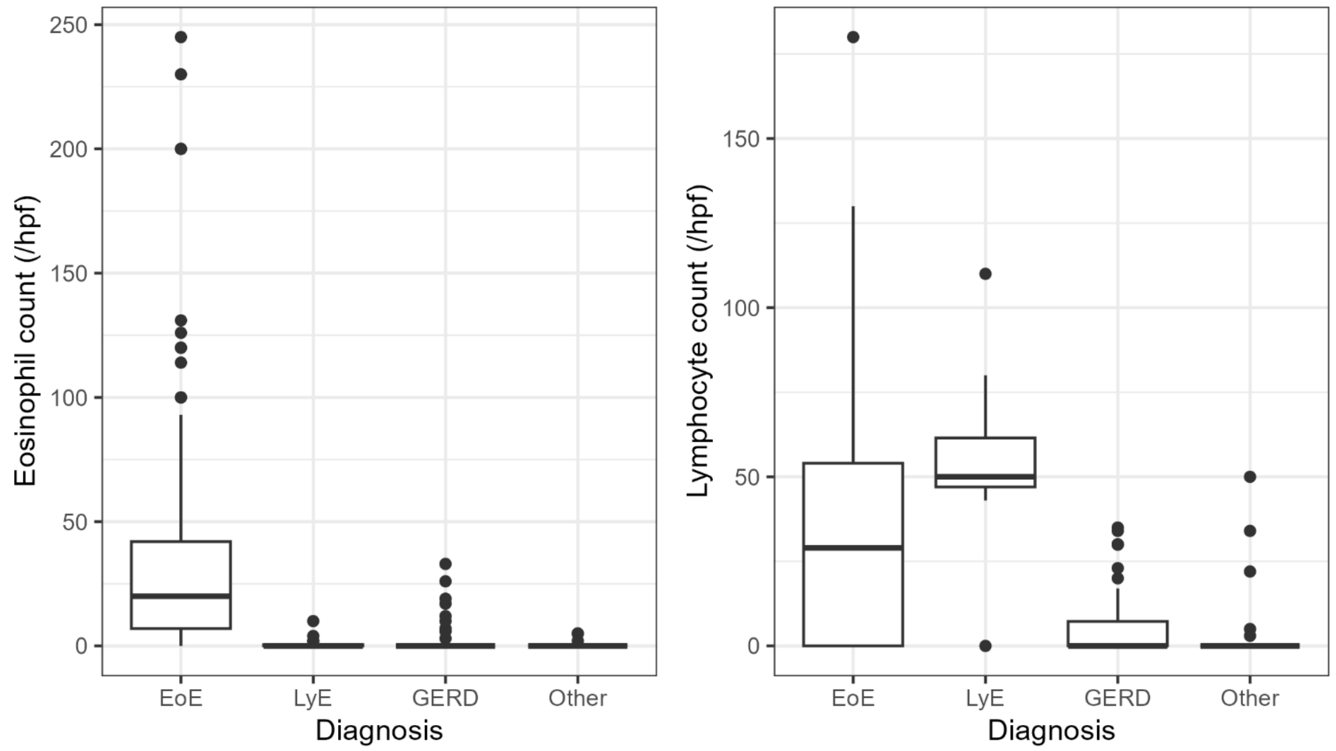

Endosc Int Open | DOI 10.1055/a-2685-7610 | © The Author(s).
